# West Syndrome Treatment: New Roads for an Old Syndrome

**DOI:** 10.3389/fneur.2013.00113

**Published:** 2013-08-12

**Authors:** Piero Pavone, Raffaele Falsaperla, Martino Ruggieri, Andrea Domenico Praticò, Lorenzo Pavone

**Affiliations:** ^1^Unit of Pediatrics and Pediatrics Emergency, University Hospital “Vittorio Emanuele,”Catania, Italy; ^2^Department of Educational Sciences, University of CataniaCatania, Italy

The recent article by Chandra et al. (1), published in *Frontiers in Neurology*, on the treatment of West syndrome (WS) by using valproate as monotherapy, prompted us to rethink about the past and present treatment strategies and the outcome in this severe epileptic syndrome.

Even though the study by Chandra et al. (1) leaves some unsolved issues regarding the lack of demonstration of hypsarrhythmia in 70% of their cases, the long-term follow-up of cognitive profiles in the whole group, an extensive etiological work-up and the alternative use of new therapeutic drugs such as vigabatrin, it provides the scientific community with successful short-term results in 36/91 (40%) of their treated WS patients who showed “…a greater than 80% decrease in the number of spasms or complete cessation.”

Historically, some previous studies in children with WS, which employed valproate as monotherapy, proved effective in controlling either hypsarrhythmia and/or the epileptic spasms (2–6). Besides valproate, zonisamide, topiramate, and nitrazepam (as monotherapy or in combination) showed good responses (ranging from 20 to 35% of treated patients) in WS (7–9). More effective, however, has proven the short-term treatment with hormonal therapy (i.e., by using the adrenocorticotropic hormone ACTH), which has been reported to succeed in 60–80% of the infants with WS treated (10). Therefore, nowadays the first-line treatment of WS and more in general of infantile spasms includes ACTH as well as vigabatrin (the latter being effective especially in infantile spasms in the setting of tuberous sclerosis) (11–13). Therefore, most recently the treatment of WS with valproate has been overtaken and overlooked (14, 15).

Overall, the treatment strategies in WS [either the first-line treatments (e.g., ACTH and vigabatrin) or the more classical non-golden treatments (e.g., valproate or other newer anticonvulsants)] are based on the assumption that an early initiation of therapy coupled with a rapid control of seizures in these patients may prevent the arrest or the decline in cognitive development.

However, the spectrum of disorders associated to *clinical spasms* with onset in infancy is wider than previously thought and is currently comprised under the umbrella term of *Infantile Spasms syndrome* (ISs), which defines an epileptic syndrome (occurring in children younger than 1 year – rarely older than 2 years), with clinical (epileptic: i.e., associated to an epileptiform EEG) spasms usually occurring in clusters whose most characteristic EEG finding is hypsarrhythmia (the spasms are often associated with developmental arrest or regression). WS refers to a form (a subset) of ISs, characterized by the combination of clustered spasms and hypsarrhythmia on an EEG. Additional (less common) phenotypes falling within the ISs include the so-called *infantile spasms single-spasm variant* (ISSV: in which spasms may occur singly rather than in clusters), *hypsarrhythmia without infantile spasms* [HWIS: in which hypsarrhythmia can be (incidentally) recorded without any evidence of clinical spasms], and *infantile spasms without hypsarrhythmia* (ISW: in which typical clinical spasms may manifest in absence of hypsarrhythmia) [reviewed in Lux and Osborne (11)]. There is a growing evidence that ISs and related phenotypes may result, besides from acquired events, from disturbances in key genetic pathways of brain development: specifically, in the gene regulatory network of GABAergic forebrain dorsal-ventral development, and abnormalities in molecules expressed at the synapse (16). Notably, children with these genetic associations also have phenotypes beyond epilepsy, including dysmorphic features, autism, movement disorders, and systemic malformations (16). In this respect, the ISs and related phenotypes, could be regarded as a (peculiar) type of neurological manifestation underlying the involvement of many different neuronal/interneuronal networks and, accordingly, the cognitive delay or arrest regarded as a (genetically predetermined) part of the (more or less severe) phenotype, which in turn is related to the type or severity of mutation or to the protein/molecule and/or regulatory network involved.

For all the above reasons, the results of Chandra et al. (1) highlight the multifaceted aspects of the spectrum of disorders associated to clinical spasms (i.e., good response to a classical anticonvulsant in the era of newer drugs and other-than valproate first-line treatments) and the prevalent role that etiological events could play in pre-programing the cognitive and behavioral outcomes in the setting of ISs (16). In this respect, without forgetting the current first-line treatment strategies for ISs and related phenotypes, we should also look with less strict therapeutic boundaries at patients with clinical spasms as in the near future the gold standard could be the development of new therapies (with new or old molecules) that simply target specific pathways of pathogenesis, as currently occurs in tuberous sclerosis (17, 18).
